# The Effect of Feed Solution Temperature on the Power Output Performance of a Pilot-Scale Reverse Electrodialysis (RED) System with Different Intermediate Distance

**DOI:** 10.3390/membranes9060073

**Published:** 2019-06-18

**Authors:** Soroush Mehdizadeh, Masahiro Yasukawa, Takakazu Abo, Masaya Kuno, Yuki Noguchi, Mitsuru Higa

**Affiliations:** 1 Graduate School of Sciences and Technology for Innovation, Yamaguchi University, 2-16-1 Tokiwadai, Ube, Yamaguchi 755-8611, Japan; soroush.mehdizadeh.70@gmail.com (S.M.); myasu@yamaguchi-u.ac.jp (M.Y.); w011fj@yamaguchi-u.ac.jp (T.A.); w023vf@gmail.com (M.K.); i047vf@yamaguchi-u.ac.jp (Y.N.); 2 Blue Energy Center for SGE Technology (BEST), Yamaguchi University, 2-16-1 Tokiwadai, Ube, Yamaguchi 755-8611, Japan

**Keywords:** reverse electrodialysis (RED), ion exchange membrane (IEM), salinity gradient energy (SGE), pilot-scale

## Abstract

Membrane-based reverse electrodialysis (RED) can convert the salinity gradient energy between two solutions into electric power without any environmental impact. Regarding the practical application of the RED process using natural seawater and river water, the RED performance depends on the climate (temperature). In this study, we have evaluated the effect of the feed solution temperature on the resulting RED performance using two types of pilot-scale RED stacks consisting of 200 cell pairs having a total effective membrane area of 40 m^2^ with different intermediate distances (200 µm and 600 µm). The temperature dependence of the resistance of the solution compartment and membrane, open circuit voltage (OCV), maximum gross power output, pumping energy, and subsequent net power output of the system was individually evaluated. Increasing the temperature shows a positive influence on all the factors studied, and interesting linear relationships were obtained in all the cases, which allowed us to provide simple empirical equations to predict the resulting performance. Furthermore, the temperature dependence was strongly affected by the experimental conditions, such as the flow rate and type of stack, especially in the case of the pilot-scale stack.

## 1. Introduction

The ever increasing energy demand worldwide and environmental issues such as CO_2_ emissions have led to an increased focus on renewable energy sources such as wind, sun and hydro power [1,2] Among all of the renewable sources used for energy production, salinity gradient energy (SGE) is known to be one of the most readily available and appropriate. SGE is defined as the electrochemical potential between two solutions with different concentrations, especially salt concentration. Theoretically, it has been estimated using the Gibbs free energy that 1.7 MJ of power may be generated when mixing 1 m^3^ of river water and a large amount of seawater. Therefore, when considering the large amount of river water discharged into seawater during the course of a year, a magnificent amount of power (1.4–2.6 TW) can be theoretically generated [3,4,5]. Reverse electrodialysis is known as a promising membrane-based process, which can directly convert SGE into an electrical current and energy [6,7,8,9,10,11,12,13,14,15,16,17]. 

**Figure 1:**
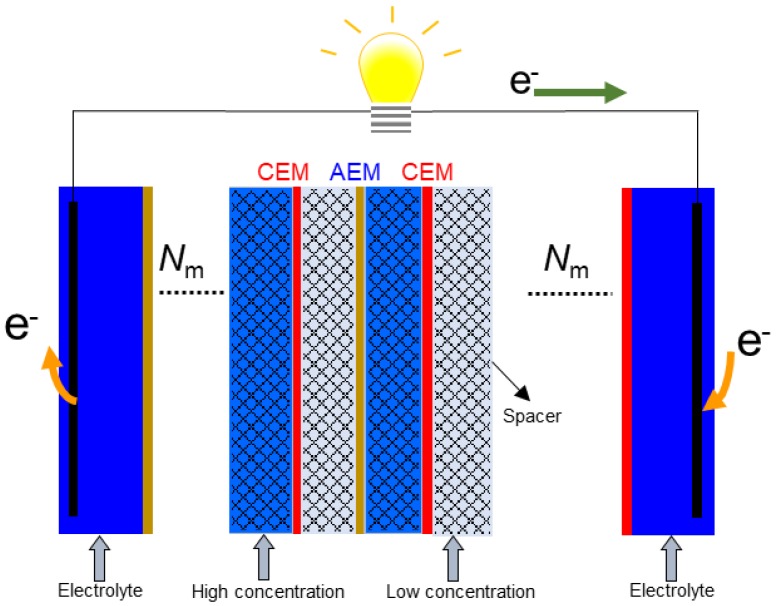
A simple schematic representation of a reverse electrodialysis (RED) stack including ion exchange membranes, integrated spacers, and electrodes.

In RED, cation exchange membranes (CEMs) as well as anion exchange membranes (AEMs) are alternatively stacked beside one another, while high and low concentration solutions flow between them, as shown in Figure 1 [18,19,20,21,22]. Integrated porous spacers are located between the membranes in order to maintain the distance between the membranes as well as playing an effective role in the solution and ion distribution. Subsequently, cations and anions are transported from the high concentration side into the low concentration side and are converted into electric current via a redox reaction using an electrode and electrolyte at both ends of the RED stack. 

Reasonably, seawater and river water have been mainly considered as the feed solutions in the RED process because they are readily available. In this case, RED power density has been varied by modifying and optimizing different parameters in the RED stack such the membrane, spacer, and operating conditions [23,24,25,26]. Among all of the operating conditions, temperature may be one of the more effective factors, which has been given less attention and could have a significant effect on RED performance [27]. Since the seaside is one of the most appropriate places for locating a RED process, the climate (temperature) of the applied solution and area will be important. Among the different areas in the world as well as the different seasons, the temperature of the feed solution may be significantly different. Increasing the temperature will lead to an increase in the solution conductivity and a decrease in the respective resistance [28]. Therefore, since the solution resistance is one of the key parameters in the RED stack resistance, temperature may have a considerable effect on the RED process performance. Although some studies have been performed to investigate the effect of temperature on the RED process performance, there are no comprehensive studies on how a pilot-scale RED process may be affected by changing the temperature. For instance, Benneker et al. showed an ~40% increase in the RED power density can be achieved by increasing the feed temperature from 20 to 40 °C using a 4-cell pair RED stack [27]. In the case of a pilot-scale RED stack, the residence time of the feed solutions in the flow channels will be higher due to the dimensions of the stack [29,30,31] and, therefore, the effect of temperature may be different. In addition, the final purpose of RED is to commercialize the process, so studying the RED process behavior on a pilot-scale will be more appropriate.

In this study, the effect of temperature on the individual feed solutions and membrane resistance was considered in order to discover the most effective parameter in the RED stack. In addition, we have investigated the effect of the temperature of the feed solutions on the power output of a pilot-scale RED system equipped with 200 and 600 µm spacers using model seawater and river water as the feed solutions. The flow rate of the feed solutions was also changed as well as the temperature to investigate their combined effects on the RED power output. 

## 2. Experimental

### 2.1. Membrane and Solution Resistance

Neosepta^®^ cation exchange membrane, CMX and anion exchange membrane, AMX (ASTOM Co., Tokyo, Japan) and the solution resistance at different temperatures were investigated using a handmade acrylic cell consisting of two parts separated by a membrane with an effective area of 1 cm^2^ according to our previous report [32]. The specific properties of CMX and AMX are shown in Table 1 [33]. The sample solution was prepared using NaCl (model seawater) with a conductivity of 49 mS/cm at 25 °C. Briefly, the sample solution was purged inside the cell and the cell was then immersed in a water bath at temperatures ranging from 10 to 35 °C to measure the solution bulk resistance without a membrane, R_bulk_. In addition, the ion conductivity of the solution at different temperatures was also measured using a conductivity meter (ES-51, HORIBA. Ltd. Tokyo, Japan). Subsequently, the same procedure was performed in the presence of a sample membrane in order to measure the resistance including both the solution and membrane resistance, R_bulk+mem_, at a particular temperature. An alternating current (AC) of 10 kHz frequency was applied to prevent an increase in the membrane resistance via the concentration polarization effect. The membrane resistance, R_mem_, was then calculated from the difference between R_bulk_ and R_bulk+mem_ as follows

(1) $$R_{\mathrm{mem}}=R_{\mathrm{bulk}+\mathrm{mem}}-R_{\mathrm{bulk}}$$

### 2.2. RED Stack

The RED experiment was performed using a pilot-scale RED stack to investigate the effect of the feed solution temperature on the RED stack performance. A 200-cell pair commercial electrodialysis stack (Acilyzer AC10-20, ASTOM Corp., Tokyo, Japan) containing commercially available ion exchange membranes (IEMs) (Neosepta® AMX and CMX) with a total membrane effective area of 40 m^2^ (each membrane area of 20 cm × 50 cm) was used. Spacers with thicknesses of 200 and 600 µm and porosity of 84 and 85%, respectively, and integrated with a gasket to prevent leakage were used. In addition, Pt-coated titanium was used as the electrode and a 5 wt.% aqueous solution of Na_2_SO_4_ was used as the electrolyte to convert ion transportation into electric current. 

### 2.3. RED Experiment

RED tests were performed using model seawater (SW) (53 ± 0.5 mS/cm NaCl aq.) and model river water (RW)/wastewater (1.3 ± 0.5 mS/cm NaCl aq.) prepared using tap water and 99.5% NaCl purchased from NACALAI TESQUE, Inc. Kyoto, Japan. The temperature of both feed solutions (SW and RW) was increased from 10 to 35 °C. The temperature and conductivity of the feed solutions were measured using a MC-31P conductive meter (DKK-TOA Corp., Tokyo, Japan). Both SW and RW were fed into the RED stack using a magnet pump (MD-30RZ-N, IWAKI CO., Ltd. Tokyo, Japan) at different flow rates (2–6 L/min). The flow rates of SW and RW were set at the same value in each experiment and the flow rate of the electrode solution was adjusted in order to maintain a low pressure difference between the feed solution compartments and the electrode solution. Therefore, the solution leakage from the feed solution compartments into the electrolyte and vice versa was negligible. Electrical performance measurements were then carried out using a PLZ 164W instrument (Kikusui electronics corp., Yokohama, Japan). Both the current (I) and voltage (V) were recorded in all the experiments using a data logging system (midi LOGGER GL200, GRAPHTEC Co., Yokohama, Japan) connected to a personal computer. I-V curve tests were performed from zero to a maximum current (until the stack voltage became zero) at a current changing rate of 0.4 mA/s. The OCV and maximum current were obtained considering the vertical and horizontal axis intercepts of the I-V curve, respectively. The RED stack resistance was also obtained from the slope of the I-V curves using Ohm’s law as follows [34]

(2) $$E_{\mathrm{stack}}=\mathrm{OCV}-R_{\mathrm{stack}}I$$

where E_stack_ and R_stack_ are the voltage and resistance of the RED stack, respectively. The RED power output, P_gross_, and power density, P_d_, are then defined using the following equations [34].

(3) $$P_{\mathrm{gross}}=E_{\mathrm{stack}}\cdot I$$

(4) Pd=PgrossNA

where N and A are the number of cell pairs and the effective membrane area of each cell, respectively. The pumping energy, P_pump_, was also calculated to consider the total net power output, P_net_, of the RED system as follows:

(5) $$P_{\mathrm{pump}}=\frac{\left({\Delta P}_{\mathrm{sea}}Q_{\mathrm{sea}}+{\Delta P}_{\mathrm{river}}Q_{\mathrm{river}}\right)}{\eta_{\mathrm{pump}}}$$

(6) $$P_{\mathrm{net}}=P_{\mathrm{gross}}-P_{\mathrm{pump}}$$

where ΔP is pressure drop within the stack, Q is the flow rate of the solution, and η_pump_ is the pumping efficiency which we assumed to be 0.85 in this study.

## 3. Results and Discussion

### 3.1. The Effect of Temperature on the Solution Resistance 

**Figure 2:**
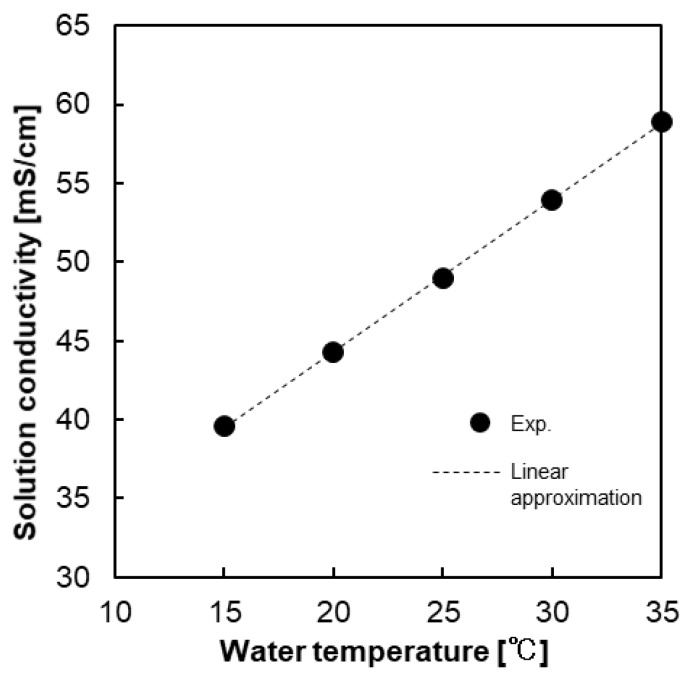
The relationship between the solution conductivity and water temperature.

The effect of temperature on the solution conductivity is shown in Figure 2. The original solution was prepared at an initial conductivity of 49 mS/cm at 25 °C and the conductivity was then measured at different temperatures ranging from 15 to 35 °C. The solution conductivity can be considered as the parameter to show the effect of temperature on the solution resistance, since the conductivity of a solution has an inverse relationship with the solution resistance (1/R). Increasing temperature enhances the ionic diffusion according the Nernst–Haskell equation because the viscosity of the solution decreases with increasing the temperature [35]. Therefore, ions can move easier upon increasing the temperature, resulting in an increase in conductivity. Moreover, conductivity increases almost linearly upon increasing temperature, and this linear relationship allows one to express the temperature dependence of the solution conductivity using the conductivity at 25 °C as a standard value as follows

(7) $$K\;\left(T\right)=K\;\left(25\;{}^{{}^{\circ}}C\right)\left[1+0.022\;\left(T-25\right)\right],\;\;\left(R^{2}=0.9998\right)$$

where K(T) and K(25 °C) are the solution conductivity (mS/cm) at temperature T (°C) and 25 °C, respectively. When considering this equation, the solution conductivity linearly increases/decreases upon increasing/decreasing the feed solution temperature with a temperature coefficient of ~2.2%/°C at 25 °C.

### 3.2. The Effect of Temperature on the Membrane Resistance

**Figure 3:**
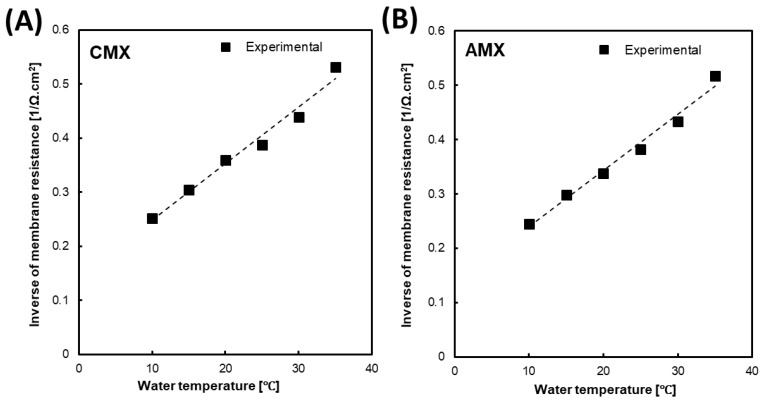
The effect of water temperature on the inverse values of the membrane resistance of CMX (**A**) and AMX (**B**).

Figure 3A,B shows the effect of the feed water temperature on the inverse of the membrane resistance (1/R_m_) of CMX and AMX, respectively, when changing the temperature from 10 to 35 °C. The ion conductivity of the membrane depends on both the ion mobility in the membrane and the ion concentration in the membrane. Donnan theory states when the ion concentration of the external solution is lower than the concentration of the fixed charged group inside the membrane, the concentration of the co-ion (ions with the same sign of charge to the fixed charged groups of membrane) is negligibly low inside the membrane and that of the counter-ions (ions with the opposite sign of charge to the fixed charged groups of membrane) is almost equal to the fixed charge groups concentration, which will be independent of the concentration of the external solution, indicating that the concentration of the counter-ions at the membrane/the external solution interface at the high concentration side is almost equal to that at the low concentration side [36]. Therefore, we assumed that the effect of feed solution concentration in the low concentration compartment on the membrane resistance is negligible. In this case, the membrane conductivity (the inverse of the membrane resistance) also increased with increasing temperature because it also leads to an increase in ion mobility in the membrane as well as in the solution. Moreover, a clear linear relationship was obtained similar to that observed with the solution conductivity. Consequently, this linear relationship provides a linear empirical equation as follows

(8) $$K_{m}\left(T\right)=K_{m}\left(25\;{}^{{}^{\circ}}C\right)\left[1+0.027\;\left(T-25\right)\right],\;\left(R^{2}=0.9800\right)\;$$

where K_m_(T) and K_m_ (25 °C) are the inverse resistance (1/R_m_) of the CMX/AMX membrane at temperature *T* and 25 °C, respectively. It is worth noting that almost the same temperature coefficient (~2.7%/°C) was obtained for both CMX and AMX. Therefore, this simple approximation may be used as an empirical equation to predict the CMX/AMX resistance at different temperatures from 15 to 35 °C. 

### 3.3. The Effect of Temperature on the RED Performance

#### 3.3.1. Open Circuit Voltage (OCV)

**Figure 4:**
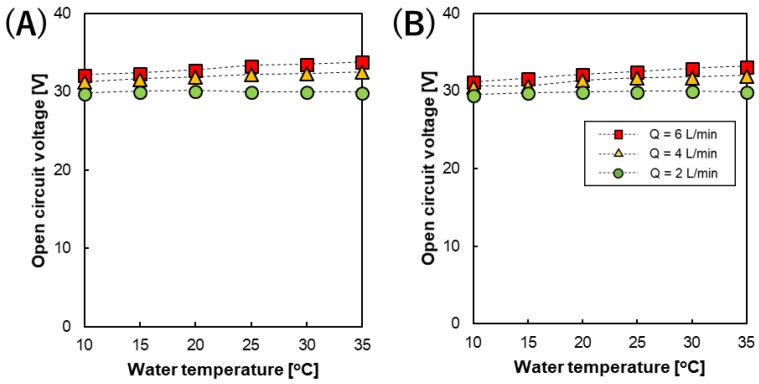
The temperature dependence of the RED stack OCV at different feed solution flow rates using an intermediate distance in the stack of (**A**) 200 and (**B**) 600 µm, respectively.

The OCV of the RED stack measured at feed flow rates (Q) of 1, 4, and 6 L/min, and at different temperatures and spacer thickness is shown in Figure 4. The highest OCVs (31.33–33.87 V) were obtained at a feed flow rate of 6 L/min in both stacks with 200 and 600 µm spacers. 

The salinity ratio between the high and low concentration compartments of the RED stack will decrease by osmotic water flow from the lower concentration side to the higher concentration side as well as co- and counter-ion diffusion from the higher to lower concentration sides during the OCV measurements, even at zero current [22]. Consequently, decreasing the salinity ratio on the membrane surface will lead to a decrease in the OCV of the RED stack. By increasing the feed flow rate, the residence time of the solution in the flow channels of the RED stack decreases, resulting in the feed solution being refreshed faster. This suppression of the decrease in the OCV was caused by water transport and ion diffusion, as mentioned above. Therefore, in our pilot-scale RED stack, the OCV increased from 29.5 V to >33 V upon increasing the feed solution flow rate from 2 to 6 L/min using both types of spacer studied. In addition, the effect of the feed solution flow rate on the OCV value in the RED stack with a 200 µm spacer was slightly higher than that using a 600 µm spacer. Actually, even when using the same flow rate, the flow velocity between the membranes were different and slightly influenced the resulting OCV value.

In addition to the feed solution flow rate, the OCV of the RED stack was also affected by temperature. Theoretically, the RED stack OCV can be defined using Equation (9), which is related to the temperature, ions valence, and concentration ratio as follows

(9) $$\mathrm{OCV}\;=\frac{N_{m}\cdot \alpha \cdot R\cdot T_{\mathrm{ab}}}{z\cdot F}\;\cdot \;\ln(\frac{C_{H}\cdot \gamma_{H}}{C_{L}\cdot \gamma_{L}})$$

where R is the gas constant, T_ab_ is absolute temperature [K], *N*_m_ is the number of cell pairs, *α* is the average membrane permselectivity, z is the valence of the ions, F is the Faraday constant, and C and γ are the concentration and activity coefficient of NaCl, respectively. Subscripts H and L represent the high and low concentration sides, respectively. Therefore, upon increasing the temperature of the feed solution, the OCV will theoretically increase with a temperature dependence of ~0.35%/°C. However, Figure 4 shows that when using a low feed solution flow rate (Q = 2 L/min), the effect of temperature on the OCV of the RED stack disappeared (0.2 and 0.4%/°C for the 200 and 600 µm cases, respectively), meaning that the OCVs at 10 and 35 °C are almost identical, while the OCV increased with a temperature dependence >2%/°C at a flow rate of 6 L/min from 10 to 35 °C in both the 200 and 600 µm spacer cases. It seems that under low feed flow rate conditions, the dominant effect on the OCV will be due to the osmotic water flow as well as co- and counter-ion transportation as mentioned above. However, upon increasing the feed flow rate, the effect of temperature on the stack OCV appears more clearly despite the presence of water and ion transportation. Empirical linear relationships between the RED stack OCV and feed solution temperature at different feed solution flow rates were also successfully obtained, as shown in Equations (10)–(12) and Equations (13)–(15) for the RED stack equipped with 200 and 600 µm spacers, respectively, in order to predict the stack OCV at different temperatures. At a flow rate of 6 L/min, 0.23%/°C and 0.26%/°C were obtained using the RED stack with 200 and 600 µm spacers, respectively, and these values were slightly less than the theoretical value (0.30%/°C) estimated using Equation (9) because of the presence of water and ion transportation, as mentioned above.

(10) $$\mathrm{OCV}\;\left(T\right)=\mathrm{OCV}\;\left(25\;{}^{{}^{\circ}}C\right)\left[1+0.0002\;\left(T-25\right)\right]\left(200\;µm\;\mathrm{at}\;2\;L/\min,R^{2}=0.0067\right)$$

(11) $$\mathrm{OCV}\;\left(T\right)=\mathrm{OCV}\;\left(25\;{}^{{}^{\circ}}C\right)\left[1+0.0015\;\left(T-25\right)\right]\left(200\;µm\;\mathrm{at}\;4\;L/\min,R^{2}=0.9742\right)$$

(12) $$\mathrm{OCV}\;\left(T\right)=\mathrm{OCV}\;\left(25\;{}^{{}^{\circ}}C\right)\left[1+0.0023\;\left(T-25\right)\right]\left(200\;µm\;\mathrm{at}\;6L/\min,{\;R}^{2}=0.9807\right)$$

(13) $$\mathrm{OCV}\;\left(T\right)=\mathrm{OCV}\;\left(25\;{}^{{}^{\circ}}C\right)\left[1+0.0004\;\left(T-25\right)\right]\left(600\;µm\;\mathrm{at}\;2L/\min,R^{2}=0.6334\right)$$

(14) $$\mathrm{OCV}\;\left(T\right)=\mathrm{OCV}\;\left(25\;{}^{{}^{\circ}}C\right)\left[1+0.0021\;\left(T-25\right)\right]\left(600\;µm\;\mathrm{at}\;4L/\min,R^{2}=0.9129\right)$$

(15) $$\mathrm{OCV}\;\left(T\right)=\mathrm{OCV}\;\left(25\;{}^{{}^{\circ}}C\right)\left[1+0.0026\;\left(T-25\right)\right]\left(600\;µm\;\mathrm{at}\;6L/\min,R^{2}=0.9922\right)$$

#### 3.3.2. RED Stack Power Output

**Figure 5:**
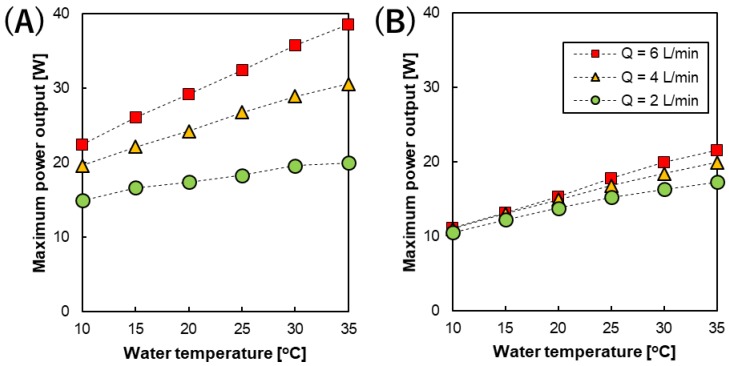
The temperature dependence of the maximum power output of the RED stack with an intermediate distance of (**A**) 200 and (**B**) 600 µm at different feed solution flow rates.

The maximum power output (gross power, P_max_) of the RED stack was measured during I–V tests conducted at different flow rates and temperatures. Figure 5A,B shows the relationship between P_max_ with different feed solution flow rates and temperatures using the RED stack equipped with 200 and 600 µm spacers, respectively. All of the I-V and I-P curves are shown in the Supplementary Information. Similar to the stack OCV, the highest P_max_ (22.5–38.6 and 11.09–21.52 W using the RED stack with 200 and 600 µm spacers, respectively) was obtained at the higher feed solution flow rate of 6 L/min, while the lower P_max_ (15–20 and 10.47–17.28 W using the RED stack with 200 and 600 µm spacers, respectively) was obtained at the lower feed solution flow rate of 2 L/min [23,37]. The higher RED stack power output observed in the 200 µm was mainly attributed to the lower resistance of the solution compartment in the stack. Moreover, linear relationships were also obtained for all the flow rates studied, although their temperature dependence was different. As a result, their linear relationships provide empirical equations that can be used to predict the gross power of the stack at different temperatures as well as different flow rates as follows.

(16) $$P_{\max}\;\left(T\right)=P_{\max}\;\left(25\;{}^{{}^{\circ}}C\right)\left[1+0.011\;\left(T-25\right)\right]\left(200\;µm\;\mathrm{at}\;2L/\min,R^{2}=0.9739\right)$$

(17) $$P_{\max}\;\left(T\right)=P_{\max}\;\left(25\;{}^{{}^{\circ}}C\right)\left[1+0.017\;\left(T-25\right)\right]\left(200\;µm\;\mathrm{at}\;4L/\min,R^{2}=0.9962\right)$$

(18) $$P_{\max}\;\left(T\right)=P_{\max}\;\left(25\;{}^{{}^{\circ}}C\right)\left[1+0.020\;\left(T-25\right)\right]\left(200\;µm\;\mathrm{at}\;6L/\min,R^{2}=0.9988\right)$$

(19) $$P_{\max}\;\left(T\right)=P_{\max}\;\left(25\;{}^{{}^{\circ}}C\right)\left[1+0.016\;\left(T-25\right)\right]\left(600\;µm\;\mathrm{at}\;2L/\min,R^{2}=0.9883\right)$$

(20) $$P_{\max}\;\left(T\right)=P_{\max}\;\left(25\;{}^{{}^{\circ}}C\right)\left[1+0.018\;\left(T-25\right)\right]\left(600\;µm\;\mathrm{at}\;2L/\min,R^{2}=0.9970\right)$$

(21) $$P_{\max}\;\left(T\right)=P_{\max}\;\left(25\;{}^{{}^{\circ}}C\right)\left[1+0.019\;\left(T-25\right)\right]\left(600\;µm\;\mathrm{at}\;6L/\min,R^{2}=0.9973\right)$$

where P_max_(T) is maximum power at temperature T and P_max_(25 °C) is the maximum power at 25 °C. The effect of temperature on the power output was significantly higher when compared with those observed for the OCV. In fact, maximum power output of RED stack is affected by both OCV and the stack resistance (R_stack_), as follows [32]

(22) $$\mathrm{Pmax}\;=\frac{V_{\mathrm{OC}}^{2}}{{4R}_{\mathrm{stack}}}$$

Actually, R_stack_ contains ohmic (membrane, solution, and electrode system resistances) and non-ohmic (concentration polarization) regimes, which are affected by both the feed solution flow rate and temperature. Increasing the feed solution flow rate is helpful to reduce the concentration polarization layer on the membrane surface as well as increasing the ion distribution, which leads to a reduction in the stack non-ohmic resistance [38,39]. On the other hand, the membrane and solution resistance decrease upon increasing the temperature due to the increase in the ion mobility and solution conductivity, as mentioned before. Therefore, upon increasing both the feed solution flow rate and temperature, the power output increased due to the reduction in the stack resistance, as shown in Figure 5. Moreover, when considering the effect of temperature on the solution and membrane resistance (Equations (7) and (8)) and the stack OCV (Equations (10)–(15)), the dominant parameters for the RED stack power output will be the solution and membrane resistance.

It is worth noting that increasing the feed solution flow rate (6 L/min) makes the power output of the RED stack become more dependent on temperature when compared to using a low feed solution flow rate (2 L/min). In fact, at a high feed solution flow rate, the stack resistance is mainly dependent on the membrane and solution resistance, while the effect of concentration polarization becomes lower. Therefore, the effect of temperature on the RED stack power output is almost in the same range as the effect of temperature on the membrane and solution resistance (2–3%/°C) when using the higher feed solution flow rate. However, in the case of the lower feed solution flow rate, the dominant parameter in the RED stack resistance is the concentration polarization, which is almost independent of temperature. Hence, at a low feed solution flow rate, the dependence of the power output of the RED stack becomes less upon changing the temperature. In addition, changing the feed solution flow rate from 2 into 6 L/min was more effective on the RED stack power output when using the 200 µm spacers. In fact, increasing the feed solution flow rate on the RED stack with the 200 µm spacer has a significant effect on the flow velocity of the feed solution between the membranes, which leads to a significant decrease in the concentration polarization effect. However, when using the RED stack with the 600 µm spacers, changing the flow rate had a lower effect on the feed velocity and subsequently, the concentration polarization effect. Since the temperature dependence with different flow rates can only be obtained using the pilot-scale experiment, the obtained results are promising toward the design of a full-scale RED system in the future.

#### 3.3.3. Pumping Energy and Net Power Output

**Figure 6:**
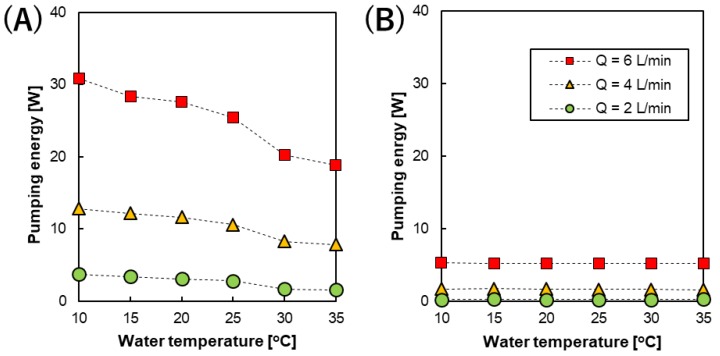
The temperature dependence of the pumping energy calculated from the pressure drop of the stack with an intermediate distance of (**A**) 200 and (**B**) 600 µm at different feed solution flow rates.

Figure 6 shows the temperature dependence of the pumping energy at different feed solution flow rates calculated from the pressure drops using Equation 5. Reasonably, the pumping energy using the 200 µm spacer was higher than that using the 600 µm spacer. This can be attributed to both the higher feed solution flow velocity and smaller intermediate distance. In addition, changing the feed solution flow rate has more of an effect on the feed velocity in the case of the RED stack with 200 µm spacers. Therefore, the difference between the pumping energy at different flow rates using the RED stack with 200 µm spacers was more prominent. 

When increasing the water temperature from 10 to 35 °C, the pumping energy gradually decreased because the viscosity of the feed solutions decreased. The viscosity of water decreases from 1.30 to 0.719 cP upon increasing the temperature from 10 to 35 °C. Furthermore, the pumping energy significantly increases at high feed solution flow rates and the smaller intermediate distance, as shown in Figure 6A. In this case, the effect of decreasing the solution viscosity became more prominent due to the higher flow velocity. Whereas, the pumping energy did not change significantly using the 600 µm spacers due to the lower feed flow velocity, as shown in Figure 6B.

**Figure 7:**
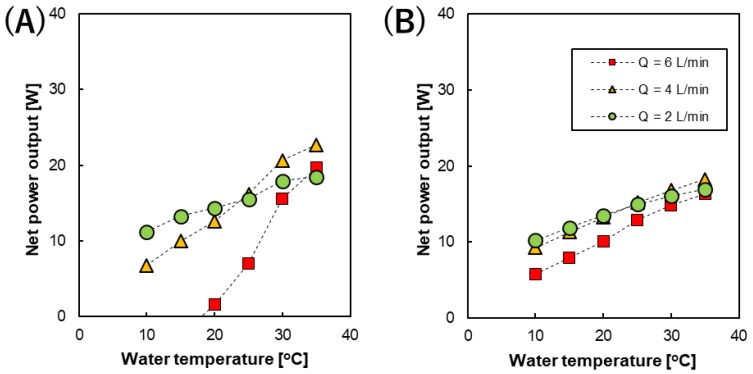
The temperature dependence of the net power output of the RED stack with an intermediate distance of (**A**) 200 and (**B**) 600 µm at different flow rates.

The net power output of the RED stack was obtained by subtracting the pumping energy from gross power output, as shown in in Figure 7. Interestingly, roughly linear relationships were still obtained under all the conditions studied although their slopes (temperature dependence) were different. The net power output of the RED stack equipped with 200 µm spacers showed steeper slopes upon increasing the temperature and the slopes rapidly changed depending on the flow rate used. On the other hand, the net power output of the RED stack with 600 µm spacers increased almost linearly with increasing temperature at all the feed solution flow rates studied, and the temperature dependence of the slopes was less steep than those observed when using 200 µm spacers. In addition, when changing the flow rate, the change in the temperature dependence was less than that observed using the 200 µm spacers. Therefore, during pilot-scale operation, conditions such as the flow rate and stack conditions (intermediate distance between the membranes) strongly influence the temperature dependence of the resulting net power output. At the highest temperature (35 °C), the RED stack with 200 µm spacers and a feed solution flow rate of 4 L/min showed the highest net power output (~22.7 W (0.57 W/m^2^)) among the all experimental conditions used in this study.

## 4. Conclusions

The effects of temperature on the solution and membrane resistance, and subsequent power generation performance of two pilot-scale RED stacks (200 and 600 µm) have been presented in this study. Both the solution and membrane resistance show linear temperature dependences of ~2.2 and 2.7%/°C, respectively. On the other hand, the temperature dependence of the RED stack OCV was ~0.2%/°C, which was independent of the intermediate distance. However, the subsequent power output and its temperature dependence were influenced by experimental conditions such as the flow rate and intermediate distance with a temperature coefficient of 1.1–2.0%/°C, which approaches its predicted value of ~3%. Furthermore, the net power output was dramatically influenced by temperature, especially in the case of the higher performance RED stack (smaller intermediate distance and high flow rate conditions). On the other hand, the temperature dependence of the resulting net power became less when using the lower performance stack (larger intermediate distance and low flow rate conditions). These results are promising for the future design of a full-scale RED system and the selection of a suitable location considering water temperature. 

## Figures and Tables

**Table 1:** Physicochemical characterization of the CMX and AMX membranes.

Membrane	Type	Thickness (mm)	Water Content (%)	Area Resistance (Ω⋅cm^2^)	Permselectivity * (%)
CMX	cation exchange membrane	0.14–0.20	25–30	1.8–3.8	97
AMX	anion exchange membrane	0.12–0.18	25–30	2.0–3.5	95

* 0.1/0.001 M NaCl at 25 °C.

**Figure A1:**
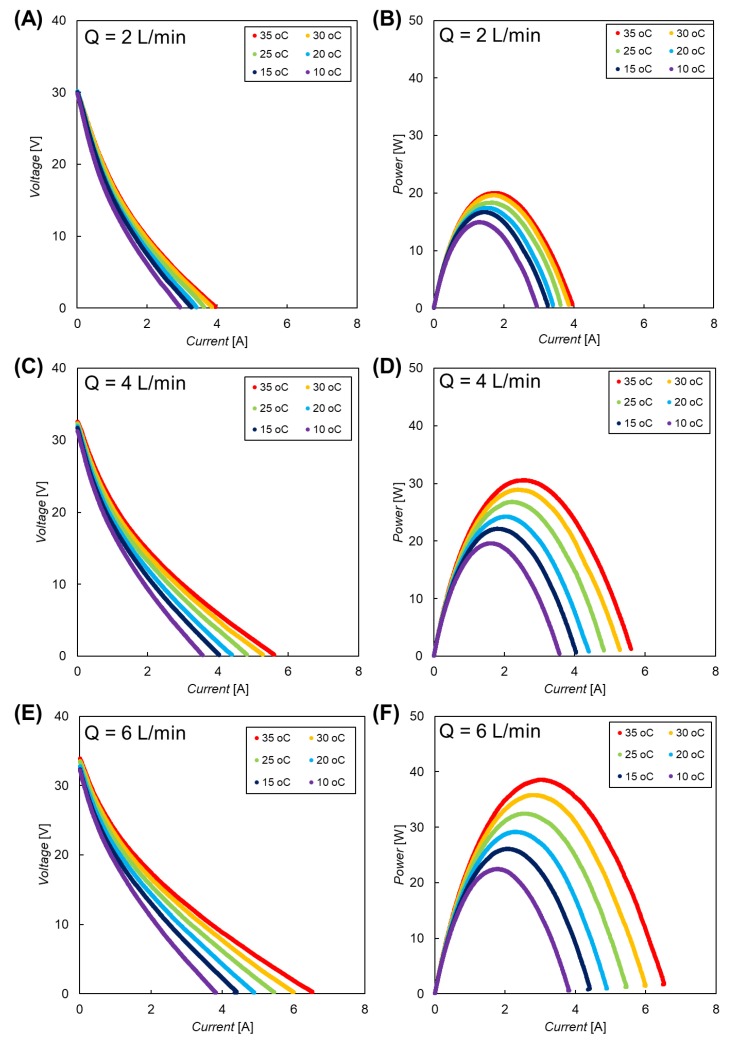
(**A**,**C**,**E**) current-voltage (I-V) and (**B**,**D**,**F**) current-power (I-P) relationships of the RED stack having the intermediate distance of 200 µm at 10–35 °C with different flow rate, Q.

**Figure A2:**
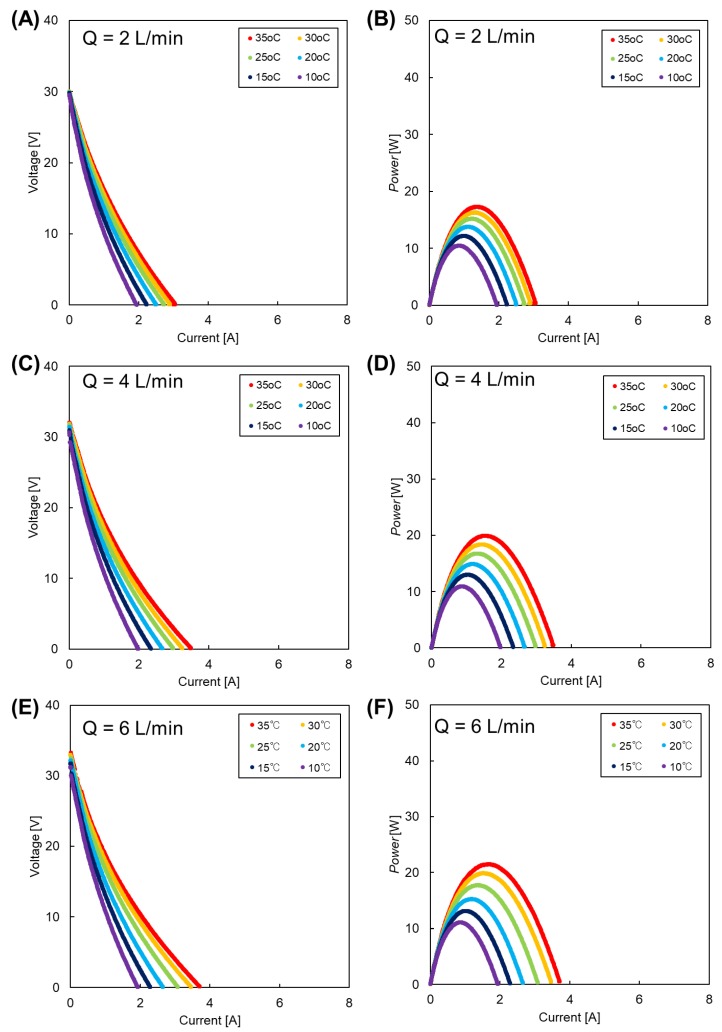
(**A**,**C**,**E**) current-voltage (I-V) and (**B**,**D**,**F**) current-power (I-P) relationships of the RED stack having the intermediate distance of 600 µm at 10–35 °C with different flow rate, Q.

## Appendix A

Figure A1 and Figure A2 show the current-voltage (I-V) and current-power (I-P) relationships of the RED stack having different intermediate distance (200 and 600 µm, respectively) at 10–35 °C with changing the flow rate. The y-intercept of I-V curve indicates the open circuit voltage (OCV) of the stack, and the maximum value of the power were subsequently used to evaluate the effect of water temperature on the resulting RED power output as shown in Figure 5.
